# Flanged intraocular lens fixation via 27‐gauge trocars using a double‐needle technique decreases surgical wounds without losing its therapeutic effect

**DOI:** 10.1111/aos.14313

**Published:** 2019-11-17

**Authors:** Hiroto Ishikawa, Hisashi Fukuyama, Yuki Komuku, Takashi Araki, Fumi Gomi

**Affiliations:** ^1^ Department of Ophthalmology Hyogo College of Medicine Nishinomiya Hyogo Japan

**Keywords:** intraocular lens implantation, intraocular lens scleral fixation, micro incision vitrectomy surgery, pars plana vitrectomy, surgery technique

## Abstract

**Purpose:**

Intraocular lens (IOL) fixation using a sutureless 27‐gauge needle intrascleral IOL implantation technique requires six surgical wounds. We developed a modified technique using two 27‐gauge trocars for vitrectomy and indwelling flanged IOL haptics to reduce the number of surgical wounds.

**Setting:**

Department of Ophthalmology, Hyogo College of Medicine.

**Design:**

This retrospective study enrolled 54 patients who had undergone IOL scleral fixation between January 2016 and April 2019.

**Methods:**

Patients who underwent IOL scleral fixation and were observed for >12 weeks were analysed using medical record data. Before October 2017, patients underwent normal flanged IOL scleral fixation. Between November 2017 and April 2019, patients underwent the modified method (flanged IOL via 27‐gauge trocars with double‐needle technique). Primary end‐point was superiority or non‐inferiority of modified IOL scleral fixation compared with normal IOL scleral fixation for visual acuity (VA). Changes in corneal endothelium cell number, refractivity, astigmatisms and surgery‐related complications, were secondary end‐points.

**Results:**

There were no significant differences in baseline characteristics between groups. Raw VA and best collected VA (BCVA) were significantly improved in all eyes (p−). There were no statistical significances in raw VA, BCVA and surgery‐related complications between groups.

**Conclusions:**

Results of the modified technique were not inferior compared with the original technique although it was less invasive. Therefore, flanged IOL fixation via trocars using a double‐needle technique is a useful technique for IOL implantation.

## Introduction

There are many reports of intraocular lens (IOL) fixation in eyes without sufficient capsular support. Representative techniques include anterior chamber IOL, iris‐fixed IOL and trans‐scleral fixed posterior chamber IOL with or without suture. In 2014, Dr. Yamane and colleagues reported sutureless 27‐gauge needle guided intrascleral IOL implantation (double‐needle technique) (Yamane et al. 2014). Since then, the IOL scleral fixation method has rapidly expanded globally.

Many retinal surgeons have attempted to evolve the IOL scleral fixation method (Akimoto et al. 2014; Ohta et al. 2014; Khan et al. 2016; Wang et al. 2016; Cutler et al. 2017; Mantopoulos et al. 2017; Thanos et al. 2017; Walsh 2017; Hu et al. 2018; John et al. 2018; Kataoka & Kamei 2018; Aaltonen et al. 2019; Bonnell et al. 2019; Gelman & Garg 2019; Hadayer et al. 2019; Sugiura et al. 2019; Walia et al. 2019; Yavuzer & Evcimen 2019) to be a minimally invasive technique. The flanged IOL scleral fixation with double‐needle technique was developed in 2019 using a 31‐gauge needle and needle stabilizer (Yamane et al. 2017, 2019); however, the number of surgical wounds required was six (one for IOL insertion, two for indwelling flanged IOL haptics and three for 27‐gauge trocars).

To investigate a minimally invasive technique, we modified the flanged IOL scleral fixation with double‐needle technique to attempt to decrease the number of surgical wounds to four by using two 27‐gauge trocars for vitrectomy and indwelling flanged IOL haptics. We compared our modified technique with the original regarding their effects on visual acuity and surgical complications.

## Materials and Methods

### Study design and eligibility

This was a retrospective study of 54 enrolled patients who had undergone IOL scleral fixation between January 2016 and April 2019. The current study was performed in accordance with the Declaration of Helsinki and with approval from the ethics committees of Hyogo College of Medicine (3236).

### Patients

Patients who had undergone IOL scleral fixation were analysed using data from medical records (observation period after surgery was greater than 12 weeks for all analysed subjects). Finally, we recruited 60 eyes from 54 patients.

### Surgical procedure

**Figure 1:**
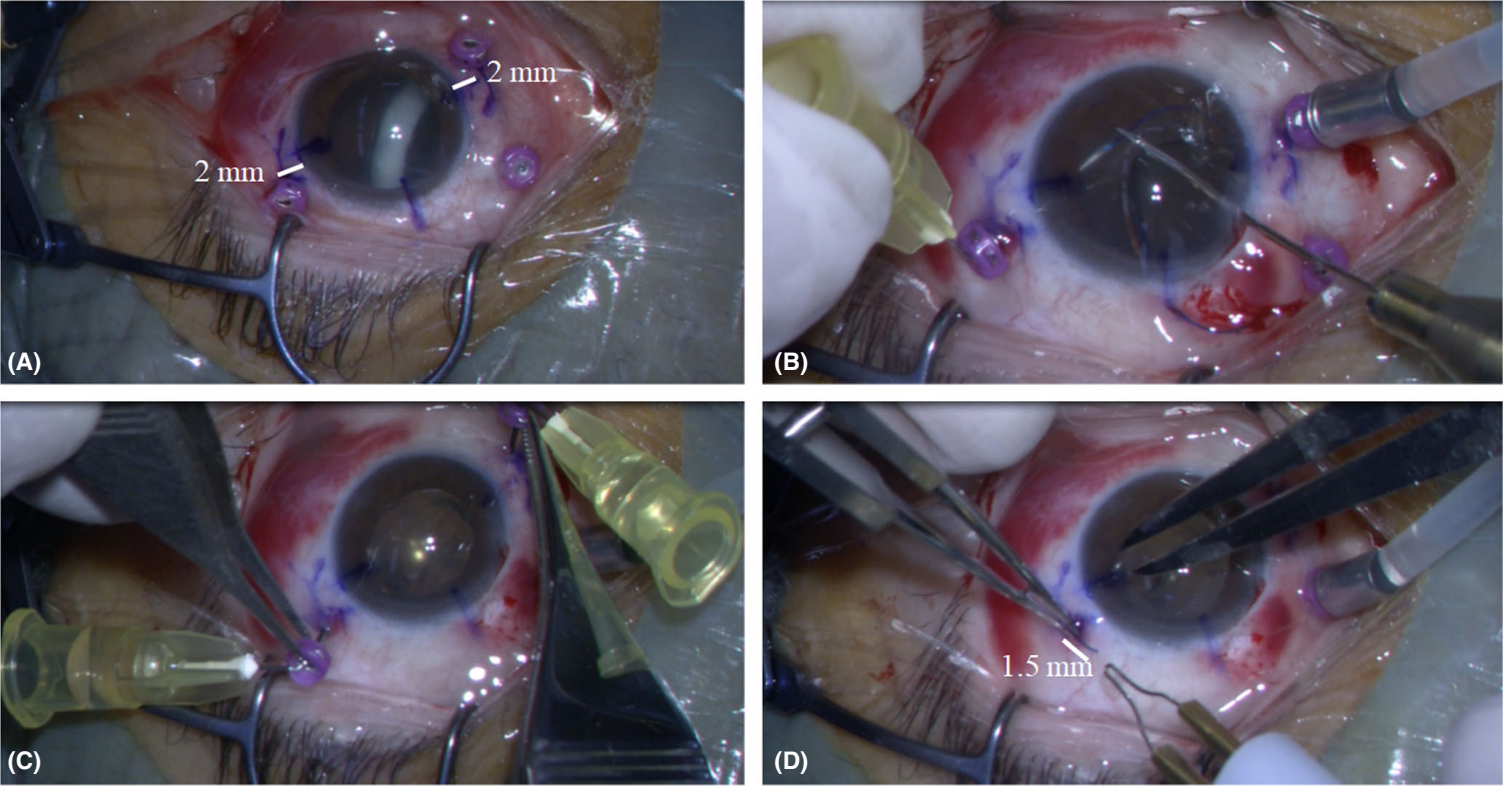
(A) 27‐gauge trocar setting: two of three trocars were set at 2 and 8 o'clock 2 mm behind the corneal limbus. (B) Intraocular lens (IOL) haptics insertion to the needle via trocar. (C) Intraocular lens scleral fixation (tip: do not pull the double needle alone, pull the double trocars with the needle). (D) Flanged IOL: irrigation length of IOL haptics was 1.5 mm.

All patients had undergone IOL scleral fixation, which was performed by a single surgeon (HI). All patients received full vitrectomy using a 27‐gauge system. Before October 2017, normal flanged IOL scleral fixation (Yamane‐flanged IOL with 27‐gauge double‐needle technique: YFD) was performed. Subsequently, a modified method (flanged IOL via 27‐gauge trocars with double‐needle technique: FvTD) was performed between November 2017 and April 2019. The modified technique was performed as follows: (1) 27‐gauge trocar setting: two of three trocars set at 2 and 8 o'clock 2 mm behind the corneal limbus, (2) full vitrectomy with or without removing dislocated lens or IOL, (3) IOL scleral fixation via two trocars using a double‐needle technique (tip: do not pull the double needle alone, pull double trocars with the needle) and (4) flanged IOL: irrigation length of IOL haptics was 1.5 mm despite 0.7 mm in the YFD group, because the scleral tunnel size formed by the 27‐gauge trocar is larger than that formed by a 27‐gauge needle, to avoid re‐dislocation of the newly inserted IOL during and after surgery (Fig. 1). Also, the surgery video is available at the online (Video S1). All IOLs were a 7.0 mm 3‐piece IOL (NX‐70; Santen, Osaka, Japan).

### Study protocol

We extracted data from medical records including: age, gender, raw and best‐corrected visual acuity (BCVA) at preoperation, 4 and 12 weeks postoperation, refractivity, lens condition before surgery, reasons for performing IOL scleral fixation (PE, eye trauma, atopic dermatitis, artificial aphakia and trouble during cataract surgery), changes in number of corneal endothelial cells, changes of astigmatism, gaps between preoperative refractive predictability and postoperative spherical equivalent power, and surgery‐related complications. Decimal VA was determined using the Landolt chart with consistent conditions: distance = 5 m and chart illuminance was 500–1000 lux. The decimal VA was then converted to log (minimum angle of resolution) (logMAR) VA as follows: logMAR = −log (decimal VA). For ‘off‐chart’ VA, such as count fingers (CF), hand motion (HM) and light perception (LP), logMAR values were set at 2.0, 2.3 and 2.6, respectively (Lange et al. 2009).

Patients were followed up for more than 12 weeks after surgery. Ophthalmological examinations were performed at postoperative weeks 4 and 12. Then, we compared changes in raw VA, BCVA, refractivity and number of corneal endothelial cells from baseline to postoperative weeks 4 and 12.

### Study end‐points

The primary end‐point was superiority or non‐inferiority of our modified technique compared with the original technique for VA. Changes of the number of corneal endothelium cells in all patients at 12 weeks between the two groups (the YFD and the FvTD), as well as refractivity, astigmatisms and surgery‐related complications, were secondary end‐points. We determined the baseline characteristics of all patients.

### Statistical analyses

For continuous variables, the mean, standard deviation (SD), standard error (SE), median and range were calculated. For discrete variables, the number of values in each category and the percentages in each category were calculated. The Student's *t*‐test or Wilcoxon signed‐rank test for continuous variables and Fisher's extract test or the Pearson chi‐squared test for categorical variables were used to assess group differences. Analyses were performed with JMP^®^ Pro (version 14.0.0, SAS Institute Inc., Cary, NC, USA). For all analyses, p‐values were reported as well as two‐sided 95% confidence intervals for point estimates. Statistical significance was determined when p‐values were <0.05.

## Results

### Baseline characteristics

Baseline characteristics in this study (*n* = 60) are shown in Table 1. Patient age (mean ± SD) was 72.1 ± 15.2 years. Patients were dominantly male (43 patients, 79.6%). The patients were separated into two groups as follows: YFD group (31 eyes, 51.7%) and FvTD group (29 eyes, 48.3%). Preoperative lens conditions were subluxated lens (20 eyes, 33.3%), IOL dislocation (30 eyes, 50.0%) and aphakia (10 eyes, 16.7%). Causative diseases were PE (19 eyes, 31.7%), trouble during cataract surgery (13 eyes, 21.7%), artificial aphakia (8 eyes, 13.3%), idiopathic (8 eyes, 13.3%) and other (12 eyes, 20.0%). There was no significant difference in age, sex, lens condition and causative diseases between these two groups. In patients with IOL dislocation, the mean time after previous cataract surgery was 12.5 ± 10.4 years.

**Table 1:** Patient characteristics at baseline (60 eyes).

	Total	YFD	FvTD	p value
Eyes	60	31	29	
Age (years)	72.1 ± 15.2 (68.2–76.0)	70.8 ± 14.3 (65.5–76.1)	73.5 ± 16.2 (67.4–79.7)	0.26
Sex	43 male/111 female	19/7	18/10	0.46
Axial length	24.56 ± 1.88	24.77 ± 1.90	24.33 ± 1.87	0.30
Preoperative lens condition				
Subluxated lens	20	14	6	0.11
IOL dislocation	30	12	18	
Aphakia	10	5	5	
Causative diseases				
PE	19	8	11	0.48
Trouble during cataract surgery	13	6	7	
Artificial aphakia	8	4	4	
idiopathic	8	6	2	
Glaucoma attack	4	3	1	
Eye trauma	4	3	1	
High myopia	2	1	1	
Atopic dermatitis	2	0	2	

FvTD = flanged IOL via 27‐gauge trocars with double‐needle technique; IOL = intraocular lens; YFD = Yamane‐flanged IOL with 27‐gauge double‐needle technique.

### Primary end‐point: efficacy of current modified intraocular lens scleral fixation for visual acuity

The raw VA and BCVA were significantly improved postoperatively in all eyes. Several parameters regarding VA are shown in Table 2. Briefly, there were no differences in pre‐ and postoperative raw VA, pre‐ and postoperative BCVA, pre‐ and postoperative spherical equivalent power and pre‐ and postoperative astigmatism of keratometry between the two groups.

**Table 2:** Changes of parameters for VA before and after surgery.

		YFD	FvTD	p value
Raw VA	Baseline	1.23 ± 0.63 (1.00 to 1.46)	1.30 ± 0.66 (1.05 to 1.56)	0.662
	4 weeks after surgery	0.72 ± 0.42 (0.57 to 0.88)	0.55 ± 0.39 (0.40 to 0.69)	0.096
	12 weeks after surgery	0.57 ± 0.34 (0.44 to 0.69)	0.45 ± 0.33 (0.32 to 0.58)	0.204
BCVA	Baseline	0.51 ± 0.69 (0.26 to 0.76)	0.47 ± 0.74 (0.19 to 0.75)	0.821
	4 weeks after surgery	0.35 ± 0.41 (0.20 to 0.5)	0.22 ± 0.28 (0.12 to 0.33)	0.168
	12 weeks after surgery	0.16 ± 0.28 (0.06 to 0.27)	0.09 ± 0.21 (0.01 to 0.18)	0.295
Spherical equivalent power	Baseline	2.46 ± 5.98 (0.18 to 4.73)	4.98 ± 6.03 (2.64 to 7.32)	0.119
	4 weeks after surgery	−0.53 ± 1.66 (−1.14 to 0.08)	−0.62 ± 1.48 (−1.18 to −0.05)	0.829
	12 weeks after surgery	−0.59 ± 1.94 (−1.30 to 0.12)	−0.28 ± 1.22 (−0.76 to 0.20)	0.477
Refractive predictability	Baseline	−0.47 ± 1.13 (−0.89 to −0.06)	−0.82 ± 1.01 (−1.20 to −0.43)	0.219
Refractive error	12 weeks after surgery	−0.12 ± 1.16 (−0.55 to −0.31)	0.46 ± 0.84 (0.13 to 0.79)	**0.031**
Astigmatism of keratometry	Baseline	−1.27 ± 0.65 (−1.50 to −1.03)	−1.70 ± 1.59 (−2.30 to −1.09)	0.171
	4 weeks after surgery	−1.35 ± 0.83 (−1.65 to −1.04)	−1.64 ± 1.29 (−2.13 to −1.15)	0.297
	12 weeks after surgery	−1.41 ± 0.96 (−1.76 to −1.06)	−1.42 ± 1.19 (−1.89 to −0.95)	0.982

Bold indicates statistical significance (p < 0.05).

BCVA = best‐corrected visual acuity; FvTD = flanged IOL via 27‐gauge trocars with double‐needle technique; IOL = intraocular lens; YFD = Yamane‐flanged IOL with 27‐gauge double‐needle technique.

### Secondary end‐points: other changes before and after surgery

The gap between postoperative spherical equivalent power and refractive predictability (reflective error) in the FvTD group (0.46 ± 0.84 dioptres) showed greater hyperopia compared with the YFD group (−0.12 ± 1.16 dioptres; p = 0.031; Table 2).

The mean number of corneal endothelial cells was 2293.5 ± 530.3 (2095.4–2491.5) in the YFD group and 2035.5 ± 636.0 (1788.9–2282.2) in the FvTD group at baseline. This significantly decreased to 1991.9 ± 530.4 (1797.4–2186.5) and 1742.6 ± 733.3 (1452.6–2032.7) postoperatively, respectively (each p−). The changes were −14.3 ± 18.7% in all eyes, −11.8 ± 19.0% in the YFD group and −17.2 ± 18.3% in the FvTD group, all of which did not reach statistical significance.

Regarding surgery‐related complications, two patients with ocular hypotension, six patients with vitreous haemorrhage (VH) and four patients with postoperative cystoid macular oedema (CME) were noted in the YFD group (12/31; 38.7%), despite one patient with ocular hypotension and two patients with VH noted in the FvTD group (3/29; 10.3%). There was no statistical significance between the groups. Patients with ocular hypotension and VH improved within a few days after surgery; however, only one case in the YFD group underwent additional surgery for protracted VH. Patients with CME received a sub‐Tenon injection of 5 mg triamcinolone acetonide, which improved their condition.

## Discussion

Intraocular lens (IOL) sutureless scleral fixation might be a convenient procedure rather than scleral fixation with suture. However, in the era of minimum invasive surgery, fewer surgical wounds would be preferable. Therefore, we developed a modified technique, flanged IOL‐double needle via trocar technique, to decrease operative stress by decreasing the number of required surgical wounds. Several studies previously reported the use of trocars for scleral fixation, but they used retinal forceps to grab the IOL haptics or suture, and not used double‐needle technique (Khan et al. 2016; Wang et al. 2016; Thanos et al. 2017; Walsh 2017; Hu et al. 2018).

Raw VA and BCVA were significantly improved postoperatively when using the original and modified technique. For the primary outcome, there were no significantly changes in all parameters regarding the VA between the YFD and FvTD groups at any timepoints. These results suggest that the FvTD technique has similar beneficial effects for VA with fewer surgical wounds when compared with YFD. This suggests our modified technique was not inferior compared with the original technique.

Regarding the secondary outcomes, the reflective error by our modified technique showed greater hyperopia compared with the original technique. Interestingly, we irrigated 1.5 mm of the IOL haptics to form flanges compared with 0.7 mm irrigation in the original technique. This suggests our modified technique might induce myopia compared with the original technique because the depth of IOL using our technique may be closer to the iris more compared with the original. However, the results suggested the opposite outcome, indicating that the scleral tunnel length made by the trocars was shorter than in the original technique, with a greater IOL depth.

Changes in the mean number of corneal endothelial cells were −14.3 ± 18.7% in all eyes, and there were no significant differences changes between the two groups. Regarding surgery‐related complications, only three eyes (10.3%) had VH and ocular hypotension when using our modified technique; however, 12 eyes (38.7%) had several complications. There were no significant differences in the incidence of these complications, supporting the idea that the technical invasion by our modified technique was not inferior compared with the original technique. In a previous report, 22% of eyes had VH, similar to the incidence of technique related complications in our study (Todorich et al. 2018).

There were several limitations in our study. First, the surgeon ‘HI’ performed over 100 cases of IOL scleral fixation using the old Yamane technique (double needle without flange IOL), then the original technique and finally our modified technique. Therefore, HI's surgical skill might have increased when performing our modified technique. Furthermore, the decrease in surgery‐related complications might be due to the surgeon's technical improvement. Second, we performed the original technique using a 27‐gauge needle before October 2017, despite Dr. Yamane using 31‐gauge needle in 2017. When we use the original technique using a 31‐gauge needle, the surgery‐related complications might be decreased. Thus, the present study did not entirely reflect the comparison between the newest Yamane technique and our modified technique.

An advantage of our modified technique is decreasing surgical wounds. Our results showed that PE was the most common reason for performing IOL fixation, suggesting that patients who received IOL fixation might also undergo glaucoma surgery in the future. Therefore, decreasing surgical wounds is very important for maintaining intact conjunctiva for further potential glaucoma surgery.

## Conclusions

Although many retinal surgeons have investigated several methods for IOL fixation, the flanged IOL‐double‐needle technique seems to have provided the best results for IOL fixation. Our modified technique was not inferior compared with the original technique of Dr. Yamane; however, the number of required wounds was reduced and therefore flanged IOL fixation via trocars using a double‐needle technique should be useful for retinal surgeons.

## Supporting information


**Video Clip S1.** Flanged IOL via 27‐gauge trocars with double‐needle technique.
