# Supplementary material for: Flanged intraocular lens fixation via 27‐gauge trocars using a double‐needle technique decreases surgical wounds without losing its therapeutic effect
Source: Acta Ophthalmol. 2019 Nov 17;98(4):e499–503. doi: 10.1111/aos.14313 (PMC7317769; doi:10.1111/aos.14313)
Supplement: Supplementary file 2 [file AOS-98-e499-s002.docx]

**Video Clip S1.** Flanged IOL via 27-gauge trocars with double-needle technique.
